# Digitalizing and Upgrading Severe Acute Respiratory Infections Surveillance in Malta: System Development

**DOI:** 10.2196/37669

**Published:** 2022-12-05

**Authors:** John Paul Cauchi, Maria-Louise Borg, Aušra Džiugytė, Jessica Attard, Tanya Melillo, Graziella Zahra, Christopher Barbara, Michael Spiteri, Allan Drago, Luke Zammit, Joseph Debono, Jorgen Souness, Steve Agius, Sharon Young, Alan Dimech, Ian Chetcuti, Mark Camenzuli, Ivan Borg, Neville Calleja, Lorraine Tabone, Charmaine Gauci, Pauline Vassallo, Joaquin Baruch

**Affiliations:** 1 Health Promotion and Disease Prevention Directorate Msida Malta; 2 Molecular Dianostics Pathology Department Mater dei Hospital Msida Malta; 3 Emergency Department Mater Dei Hospital Msida Malta; 4 Mater Dei Hospital Msida Malta; 5 Directorate for Health Information and Research Msida Malta; 6 Primary Health Care Ministry of Health Floriana Malta; 7 Superintendent of Public Health Ministry of Health Msida Malta; 8 European Programme for Intervention Epidemiology Training program European Centre for Disease Prevention and Control Solna Sweden

**Keywords:** surveillance, public health, epidemiology, COVID-19, disease prevention, disease surveillance, digital health, health system, pandemic, public hospital, patient data, health data, electronic record, monitoring

## Abstract

**Background:**

In late 2020, the European Centre for Disease Prevention and Control and Epiconcept started implementing a surveillance system for severe acute respiratory infections (SARI) across Europe.

**Objective:**

We sought to describe the process of digitizing and upgrading SARI surveillance in Malta, an island country with a centralized health system, during the COVID-19 pandemic from February to November 2021. We described the characteristics of people included in the surveillance system and compared different SARI case definitions, including their advantages and disadvantages. This study also discusses the process, output, and future for SARI and other public health surveillance opportunities.

**Methods:**

Malta has one main public hospital where, on admission, patient data are entered into electronic records as free text. Symptoms and comorbidities are manually extracted from these records, whereas other data are collected from registers. Collected data are formatted to produce weekly and monthly reports to inform public health actions. From October 2020 to February 2021, we established an analogue incidence-based system for SARI surveillance. From February 2021 onward, we mapped key stakeholders and digitized most surveillance processes.

**Results:**

By November 30, 2021, 903 SARI cases were reported, with 380 (42.1%) positive for SARS-CoV-2. Of all SARI hospitalizations, 69 (7.6%) were admitted to the intensive care unit, 769 (85.2%) were discharged, 27 (3%) are still being treated, and 107 (11.8%) died. Among the 107 patients who died, 96 (89.7%) had more than one underlying condition, the most common of which were hypertension (n=57, 53.3%) and chronic heart disease (n=49, 45.8%).

**Conclusions:**

The implementation of enhanced SARI surveillance in Malta was completed by the end of May 2021, allowing the monitoring of SARI incidence and patient characteristics. A future shift to register-based surveillance should improve SARI detection through automated processes.

## Introduction

### Severe Acute Respiratory Infections Surveillance

In 2020, the European Centre for Disease Prevention and Control (ECDC) invited countries across Europe to collaborate in setting up a surveillance system for patients with severe acute respiratory infections (SARI). In general terms, a patient with SARI is defined as a patient who is hospitalized presenting with acute respiratory symptoms (see more details below). Public health surveillance systems are vital tools to monitor the incidence and severity of infectious diseases in a population [1,2]. Most European countries had already set up a surveillance system monitoring influenza-like illness and regularly report to the European Influenza Surveillance Network [3].

With the onset of COVID-19, an international surveillance program (European SARI Network [E-SARI-Net]) was rolled out in the World Health Organization (WHO) European Region, focusing on SARI and encouraging European collaboration between public health authorities while also monitoring known and novel illnesses, such as influenza and COVID-19. The primary objectives of this international surveillance program are as follows: (1) to monitor incidence trends and describe SARI cases by etiology and demographics; (2) to describe the intensity and activity of SARI; (3) to identify, describe, and monitor at-risk groups for severe disease; and (4) to detect unusual and unexpected events (eg, new pathogens). Secondary objectives are as follows: (1) to assess the impact of SARI on health systems and the impact of interventions on SARI incidence and (2) to assess and analyze the SARI burden of disease.

The rollout and establishment of the surveillance system were supervised by Epiconcept [4], who liaised with existing and newly established SARI surveillance teams in several countries. In the E-SARI-Net, countries were invited to collect questionnaire-based or register-based data. A questionnaire-based system obtains information directly from patients with SARI or from their medical notes, whereas a register-based system collects data directly entered into registers, which can then be extracted for monitoring and analysis. Questionnaire-based systems can be rolled out and updated easily; however, they require staff and time. In some countries such as Germany, where SARI surveillance has been established successfully for many years, register-based surveillance is the preferred data collection method as it is easily extractable, fast, and requires minimal investment in staff [5].

### Background Information in Malta

**Figure 1:**
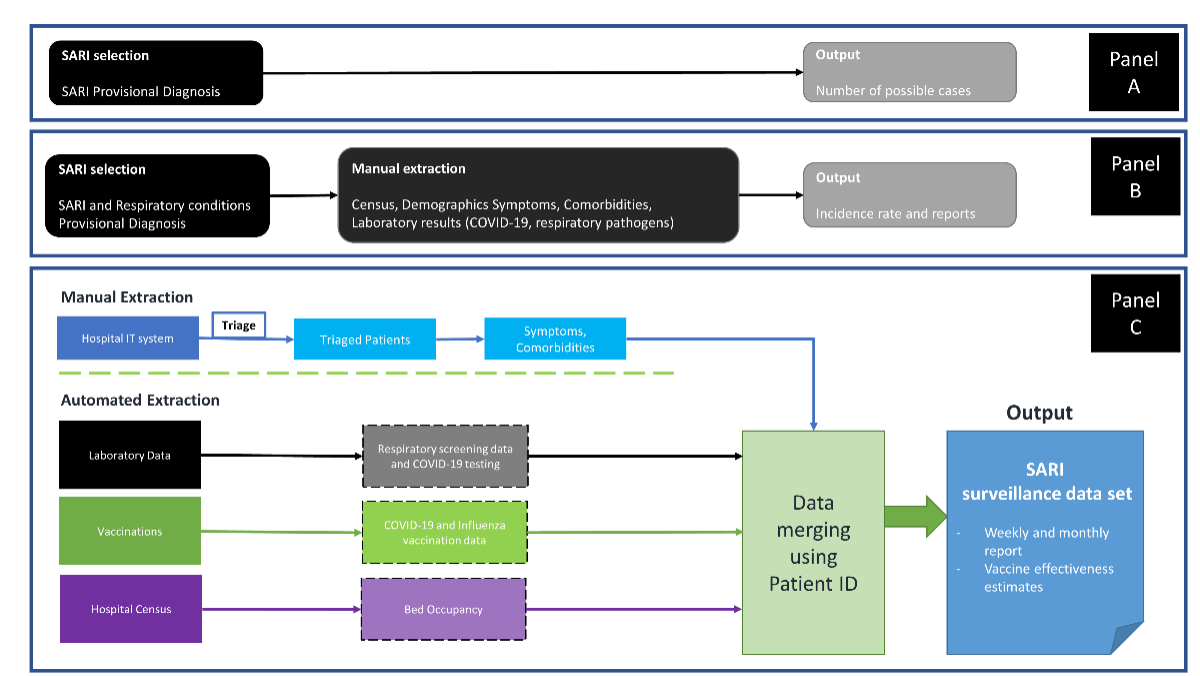
Flowchart describing SARI surveillance procedures in Malta. Panel A shows the system in place in October 2020. Panel B shows the system in place from February 1, 2021, which included data required by the ECDC protocol. Panel C shows the system currently in place, where manual extraction is required only for symptoms and comorbidities. ECDC: European Centre for Disease Prevention and Control; SARI: severe acute respiratory infections.

Malta is a central Mediterranean island country and the smallest country in the European Union. It had a population of 516,100 at the end of 2020 [6], giving it one of the world’s highest population densities at over 1376 people/km^2^ [7]. All SARI cases in Malta are admitted to a central hospital: Mater Dei Hospital (MDH). Over the course of the last 2 and a half years, the following milestones occurred. (1) Before the pandemic, patient records were paper-based and stored in folders, which made data extraction nearly impossible, with access restricted to those with physical access to those files. (2) Since the start of the COVID-19 pandemic in Malta (March 2020), MDH has established and improved its capacity to carry out surveillance and data accessibility, with hospital staff entering medical admission notes electronically as free text in an accessible database. (3) Since October 19, 2020, the hospital started identifying SARI hospitalized cases based on a provisional diagnosis provided by clinicians. Through this process, an initial incidence-based system was put in place (Figure 1, Panel A).

In November 2020, Malta accepted to be part of the E-SARI-Net. Throughout this paper, we narrate the process of setting up a comprehensive SARI surveillance system in Malta and describe its output, along with challenges that we encountered and future steps in improving the system. We will also discuss the advantages and disadvantages of different SARI case definitions.

## Methods

### Study Details

This study describes the process of digitizing and upgrading SARI surveillance in Malta, an island country with a centralized health system, during the COVID-19 pandemic, focusing on SARI surveillance from February 1 to November 30, 2021. As aforementioned, Malta has gone from a paper-based system to one that is increasingly digitized, allowing the establishment of syndromic public health surveillance. This process is described in Figure 1. The results section includes descriptive data and a comparison of COVID-19 detection outcomes between different SARI definitions.

### Definitions of Patients With SARI and COVID-19

The definition of a patient with SARI differs from one organization to another but holds key standard identifiers, with a patient with SARI being:

1. a patient with an acute respiratory condition, who has been
2. hospitalized for more than 24 hours, with
3. the onset of symptoms ≥10 days prior to admission, and presenting with
4. specific symptoms (see below).

The WHO SARI definition includes the following symptoms [8]: history of fever or measured fever of ≥38 °C and cough. The ECDC, although not having a specific definition for SARI, has a specific definition for patients with COVID-19—any person with at least one of the following symptoms: cough; fever; shortness of breath; and sudden onset of anosmia, ageusia, or dysgeusia [9].

In Malta, we have included these common criteria and the following symptoms to define a patient with SARI:

- Fever (>38.0 °C) OR feverishness (37.0 °C to 37.9 °C or reported history of fever) AND
- Acute onset of cough OR shortness of breath

This is a hybrid of WHO’s [8] SARI definition and the ECDC definition of a patient who is COVID-19 positive [9]. The definition of a patient with COVID-19 follows the protocol established by the ECDC [9] and international guidelines: a positive case of COVID-19 is a patient who tested positive for the SARS-CoV-2 virus by polymerase chain reaction either during the first 2 days of hospitalization or up to 14 days prior to hospitalization.

To compare the WHO definition and our hybrid definition, we evaluated the percentage of patients with SARI who are COVID-19 positive that met our criteria by month and age category, among those eligible for triage (see below).

### Establishing an SARI Surveillance System

#### Creating the Team

After signing the agreement with Epiconcept in late November 2020, data started being collected (see Multimedia Appendix 1 for the variables collected), and we established an SARI surveillance team. This team was comprised of a medical doctor, an EPIET fellow, and a local public health consultant; this team expanded over time. This team examined the ECDC protocol and commenced a mapping exercise of the key stakeholders and data sources.

#### Mapping Key Stakeholders

Until recently, Malta had limited efforts to consolidate data into a centralized system easily accessible for public health analysis. Consequently, a mapping exercise for critical stakeholders was undertaken: for example, contacting the vaccination team stakeholder, organizing a meeting with the team, and seeking permission to obtain their data. This exercise, although time-consuming, was beneficial in the long run as it brought together different expertise working together for a common goal—the establishment of the SARI surveillance system.

#### Manual Extraction of Data

On February 1, 2021, we started extracting data from the system that had been established in October 2020 (Figure 1, Panel A), this time including the data required by the ECDC protocol (Figure 1, Panel B). Here, clinical diagnoses provided by doctors specifically about either “SARI” or “Respiratory conditions” were flagged and then triaged according to the SARI definition above. Patients assigned a “SARI” provisional diagnosis were included automatically and classified as “confirmed” if they met the case definition or “probable” if they did not. Patients assigned a “Respiratory conditions” provisional diagnosis were only included if they met the case definition and were therefore all “confirmed.” The distinction between “confirmed” and “possible” cases will be discussed further below. Past triage notes written by the doctor were read and their data were entered into a Microsoft Excel file to store the data. This required the manual extraction of data, which was time-consuming, as data had to be sourced from free-text entries in multiple different data sets. This system was still inefficient for surveillance, requiring the reading of patient records. However, it was an essential first step that permitted establishing a surveillance system.

A patient categorized as “hospitalized” is defined as a patient who has been admitted to hospital for more than 24 hours, as per WHO SARI protocol. Additionally, patient outcomes were also categorized in order of severity: hospitalized, admitted to intensive care unit (ICU), and death. Patients in the ICU and those who died were considered within the hospitalized cohort, and their counts in sections below are nested within the hospitalized cohort. Information on ICU admission was obtained from the daily hospital bed census, and death outcomes were obtained from the mortality register.

#### A Step Toward Automation: a Hybrid System

Currently, symptoms and comorbidities are collected from the IT system of the hospital’s accident and emergency admissions department as described above. However, the rest of the information are collected through a merging of data sets (Figure 1, Panel C). Collected data are cleaned and matched using patient identification numbers to vaccination data (COVID-19 and influenza); laboratory data (COVID-19, influenza, and a respiratory panel of 32 pathogens); and the hospital census (to identify the patient’s journey through hospital). The process of data cleaning and merging is carried out using R statistical software (version 4.1; R Foundation for Statistical Computing) [10], with scripts frequently updated to deal with the dynamic developments in the surveillance system.

### Ethical Consideration

No ethical protocols are required in Malta for surveillance systems as long as data are anonymized and aggregated.

## Results

### Patient Demographics

**Figure 2:**
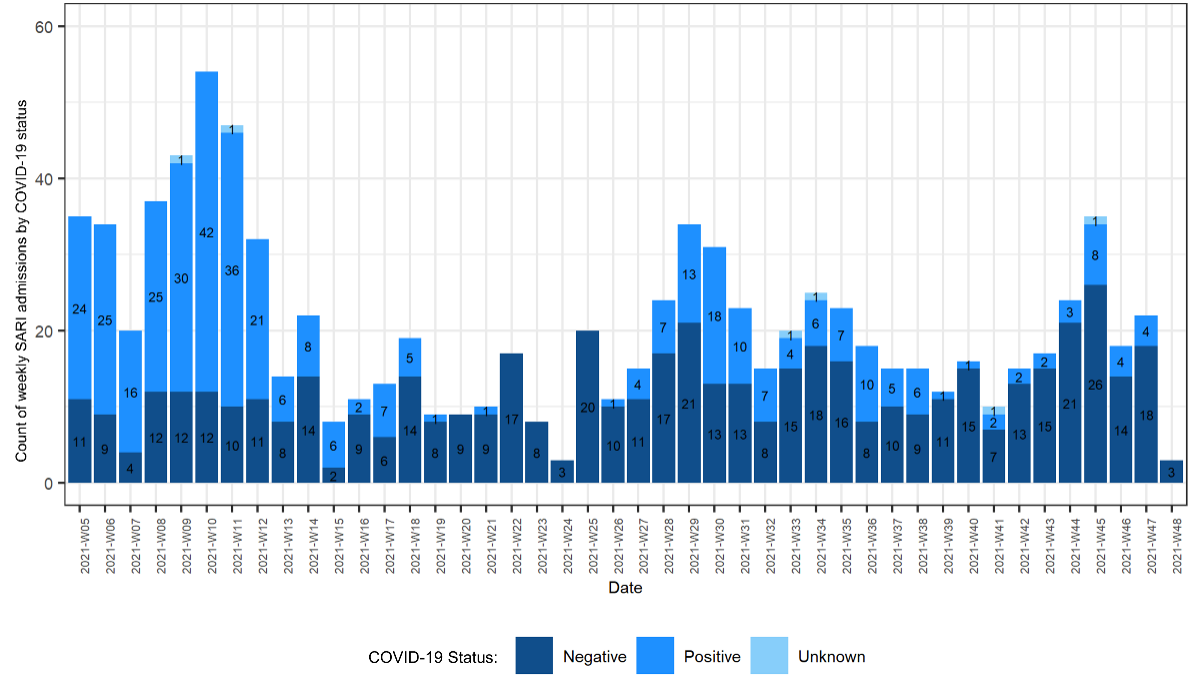
Weekly SARI case counts according to COVID-19 status. SARI: severe acute respiratory infections.

From February 1 to November 30, 2021, 903 patients with SARI were detected. Figure 2 shows the epidemiological curve of weekly SARI admissions. The COVID-19 status of patients is shown in different colors; the proportion of patients who are COVID-19 positive matched the COVID-19 “wave” that occurred from March to April 2021 and the following, smaller COVID-19 “wave” in July 2021.

Table 1 describes the demographics and characteristics of people included in the surveillance system, including symptoms, underlying conditions, clinical conditions, and outcomes, grouped according to worsening severity of symptoms (from least to most severe: hospitalization, admission to ICU, death). It is important to note that those admitted to the ICU and those who died are nested within the hospitalized cohort and are not separate from it. Among the 903 cases, 380 (42.1%) were COVID-19 positive, and 107 (11.8%) died in hospital. In all, 615 (68.1%) patients were aged >60 years, and 531 (58.8%) were male. Among the 107 patients who died, 96 (89.7%) had more than one underlying condition, the most common of which were hypertension (n=57, 53.3%) and chronic heart disease (n=49, 45.8%).

**Figure 3:**
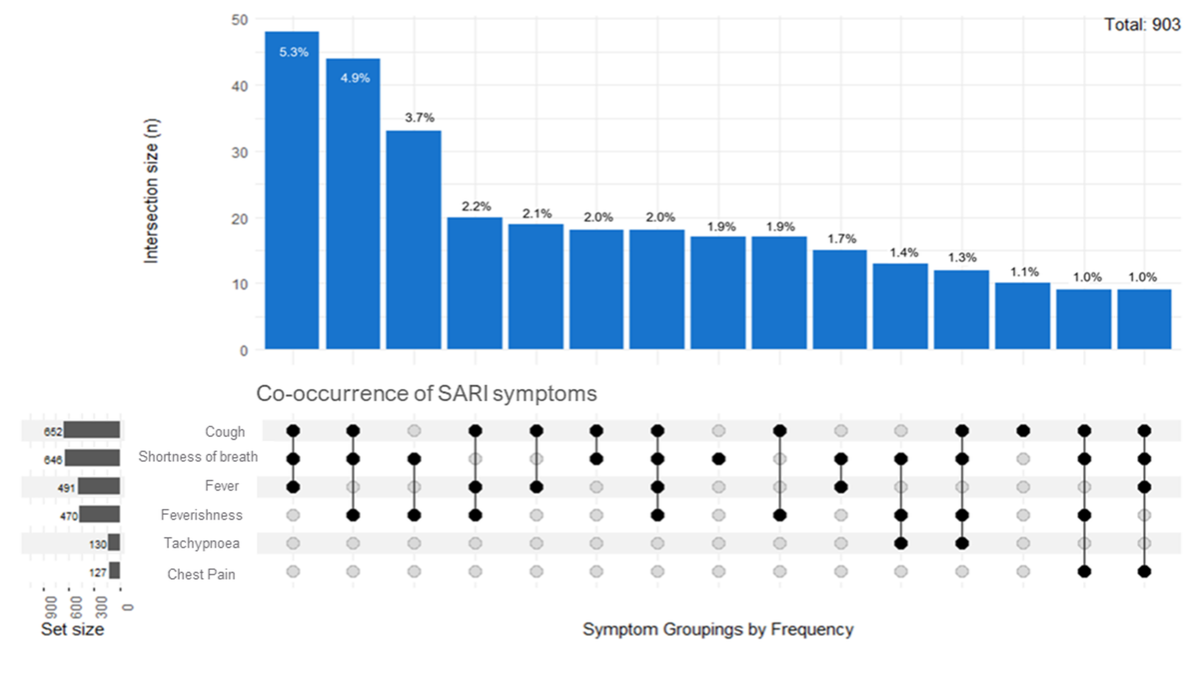
Upset plot showing the most common symptom groupings for patients with severe acute respiratory infections (SARI).

Figure 3 shows the combination of SARI symptoms for confirmed SARI cases (n=735). The most common combination of symptoms is cough, shortness of breath, and fever, with 5.4% (49/903) of patients displaying all 3 symptoms concurrently. Other common co-occurring symptoms are tachypnea and chest pain.

**Table 1:** Demographics and characteristics of patients with SARI^a^ by severity categories, in Malta from February 1, 2021, to November 30, 2021.

Characteristic		Hospitalized (N=903)	ICU^b^ admission (n=69)	Death (n=107)
Total in hospitalized category (N=903), n (%)^c^		903 (100)	69 (7.6)	107 (11.8)
Status, confirmed SARI cases^d^, n (%)		735 (81.4)	52 (75.4)	80 (74.8)
**Sex, n (%)**				
	Male	531 (58.8)	53 (76.8)	64 (59.8)
	Female	369 (40.9)	16 (23.2)	43 (40.2)
Age (years), median (IQR)		69.5 (55-80)	63.0 (54-71)	78.0 (70-85)
**Age group (years), n (%)**				
	<21	5 (0.6)	0 (0)	0 (0)
	21-40	101 (11.2)	6 (8.7)	1 (0.9)
	41-60	179 (19.8)	24 (34.8)	9 (8.4)
	61-80	403 (44.6)	38 (55.1)	51 (47.7)
	81+	212 (23.5)	1 (1.4)	46 (43)
**Symptoms, n (%)**				
	Altered taste	14 (1.6)	0 (0)	0 (0)
	Anosmia	28 (3.1)	3 (4.3)	2 (1.9)
	Cough	651 (72.1)	56 (81.2)	65 (60.7)
	Fever	488 (54)	44 (63.8)	49 (45.8)
	Feverishness	470 (52)	23 (33.3)	58 (54.2)
	Loss of taste	26 (2.9)	1 (1.4)	1 (0.9)
	Malaise	26 (2.9)	3 (4.3)	1 (0.9)
	Nausea	100 (11.1)	6 (8.7)	3 (2.8)
	Shortness of breath	644 (71.3)	55 (79.7)	78 (72.9)
	Vomiting	113 (12.5)	7 (10.1)	10 (9.3)
**Underlying conditions, n (%)**				
	More than one underlying condition	854 (94.6)	65 (94.2)	96 (89.7)
	Asthma	85 (9.4)	5 (7.2)	5 (4.7)
	Chronic heart disease	269 (29.8)	13 (18.8)	49 (45.8)
	Chronic lung disease	87 (9.6)	2 (2.9)	18 (16.8)
	Chronic kidney disease	61 (6.8)	7 (10.1)	8 (7.5)
	Cancer	104 (11.5)	4 (5.8)	23 (21.5)
	Diabetes	230 (25.5)	25 (36.2)	28 (26.2)
	Dyslipidemia	111 (12.3)	9 (13)	15 (14)
	Hypertension	394 (43.6)	32 (46.4)	57 (53.3)
	Obese	43 (4.8)	8 (11.6)	6 (5.6)
**Clinical conditions, n (%)**				
	Acute respiratory distress syndrome	36 (4)	13 (18.8)	6 (5.6)
	Pneumonia	662 (73.6)	63 (91.3)	58 (54.2)
**Outcome, n (%)**				
	Discharged	769 (85.2)	46 (66.7)	N/A
	Died	107 (11.8)	20 (30)	107 (100)
	Still being treated	27 (3)	3 (4.3)	N/A

^a^SARI: severe acute respiratory infections.

^b^ICU: intensive care unit.

^c^This row shows row percentages, whereas other rows show column percentages.

^d^“Confirmed” SARI cases are patients who matched the SARI triage definition; these are in contrast to “possible” SARI cases, which are patients diagnosed as having SARI by the doctor who had missing symptomatic data provided.

### Patients Who Are COVID-19 Positive

Although SARI surveillance is not exclusive to COVID-19, it was a substantial cause for SARI admissions during the period described here. Table 2 shows the characteristics of patients with SARI testing positive for SARS-CoV-2 (n=380). COVID-19 accounted for 42.2% (380/900) of all SARI hospitalizations, 86.9% (60/69) of all SARI ICU admissions, and 41.1% (44/107) of SARI deaths.

**Table 2:** Demographics and characteristics of patients with SARI^a^ who are COVID-19 positive by severity categories, in Malta from February 1, 2021, to November 30, 2021, excluding those with unknown COVID-19 status (n=3). Total number of patients included in this table is 900.

Characteristic			Hospitalized (n=380)		ICU^b^ admission (n=60)		Death (n=44)
COVID-19 positive, n/N (%)^c^			380/900 (42.2)		60/69 (86.96)		44/107 (41.1)
**Sex, n (%)**							
	Male	244 (64.2)		45 (0.75)		30 (68.2)	
	Female	136 (35.8)		15 (0.25)		14 (31.8)	
Age (years), median (IQR)			69.5 (55-80)		63.0 (54-71)		78 (70-85)
**Age group (years), n (%)**							
	<21	4 (1.1)		0 (0)		0 (0)	
	21-40	57 (15)		4 (6.7)		0 (0)	
	41-60	104 (27.4)		20 (33.3)		3 (5)	
	61-80	179 (47.1)		35 (58.3)		26 (59.1)	
	81+	36 (9.5)		1 (1.7)		15 (34.1)	
**Symptoms, n (%)**							
	Altered taste	13 (3.4)		0 (0)		0 (0)	
	Anosmia	25 (6.6)		3 (5)		2 (4.5)	
	Cough	285 (75)		50 (83.3)		30 (68.2)	
	Fever	192 (50.5)		39 (65)		18 (40.9)	
	Feverishness	167 (43.9)		21 (35)		21 (47.7)	
	Loss of taste	24 (6.3)		1 (1.7)		1 (2.3)	
	Malaise	15 (3.9)		3 (5)		0 (0)	
	Nausea	60 (15.8)		6 (10)		1 (2.3)	
	Shortness of breath	263 (69.2)		47 (78.3)		30 (68.2)	
	Vomiting	49 (12.9)		5 (8.3)		1 (2.3)	
**Underlying conditions, n (%)**							
	Asthma	32 (8.4)		4 (6.7)		4 (9.1)	
	Cancer	23 (6.1)		4 (6.7)		7 (15.9)	
	Chronic heart disease	68 (17.9)		11 (18.3)		22 (50)	
	Chronic lung disease	12 (3.2)		1 (1.7)		4 (9.1)	
	Chronic kidney disease	21 (5.5)		6 (10)		5 (11.4)	
	Diabetes	98 (25.8)		22 (36.7)		14 (31.8)	
	Dyslipidemia	48 (12.6)		7 (11.7)		5 (11.4)	
	Hypertension	140 (36.8)		27 (45)		30 (68.2)	
	Obese	19 (5)		8 (13.3)		4 (9.1)	
**Clinical conditions, n (%)**							
	Acute respiratory distress syndrome	18 (4.7)		11 (18.3)		3 (6.8)	
	Pneumonia	348 (91.6)		55 (91.7)		31 (70.5)	

^a^SARI: severe acute respiratory infections.

^b^ICU: intensive care unit.

^c^This row shows row percentages, whereas other rows show column percentages.

### Comparing Malta’s SARI Definition With the WHO SARI Definition

**Figure 4:**
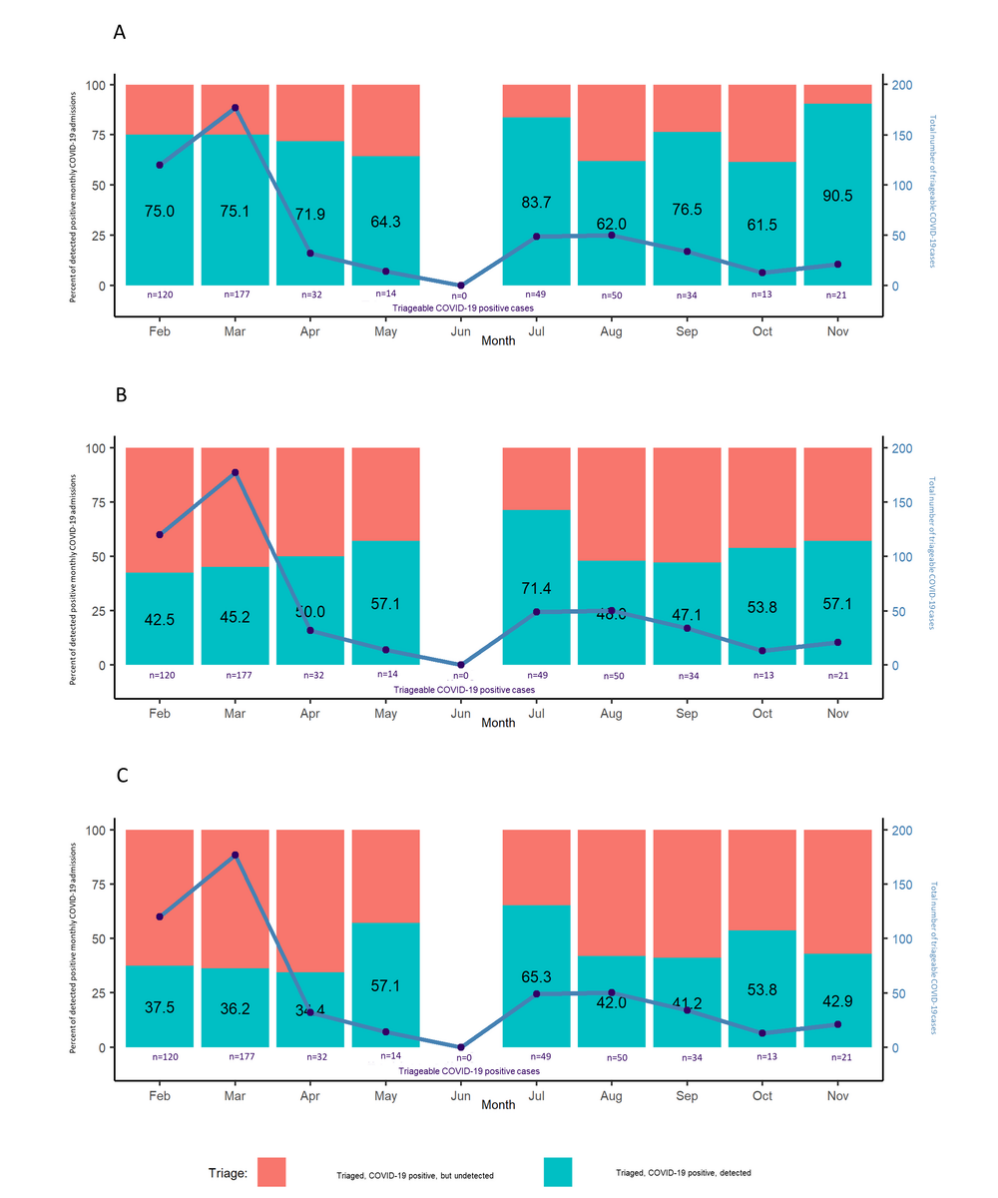
Comparing SARI definitions: patients with COVID-19 detected by month from February 1 to November 30, 2021. Percent of patients who are COVID-19 positive detected by triage using (A) Maltese SARI "confirmed" and "possible" definitions, (B) Maltese SARI "confirmed" definition, and (C) the World Health Organization's definition. SARI: severe acute respiratory infections.

**Figure 5:**
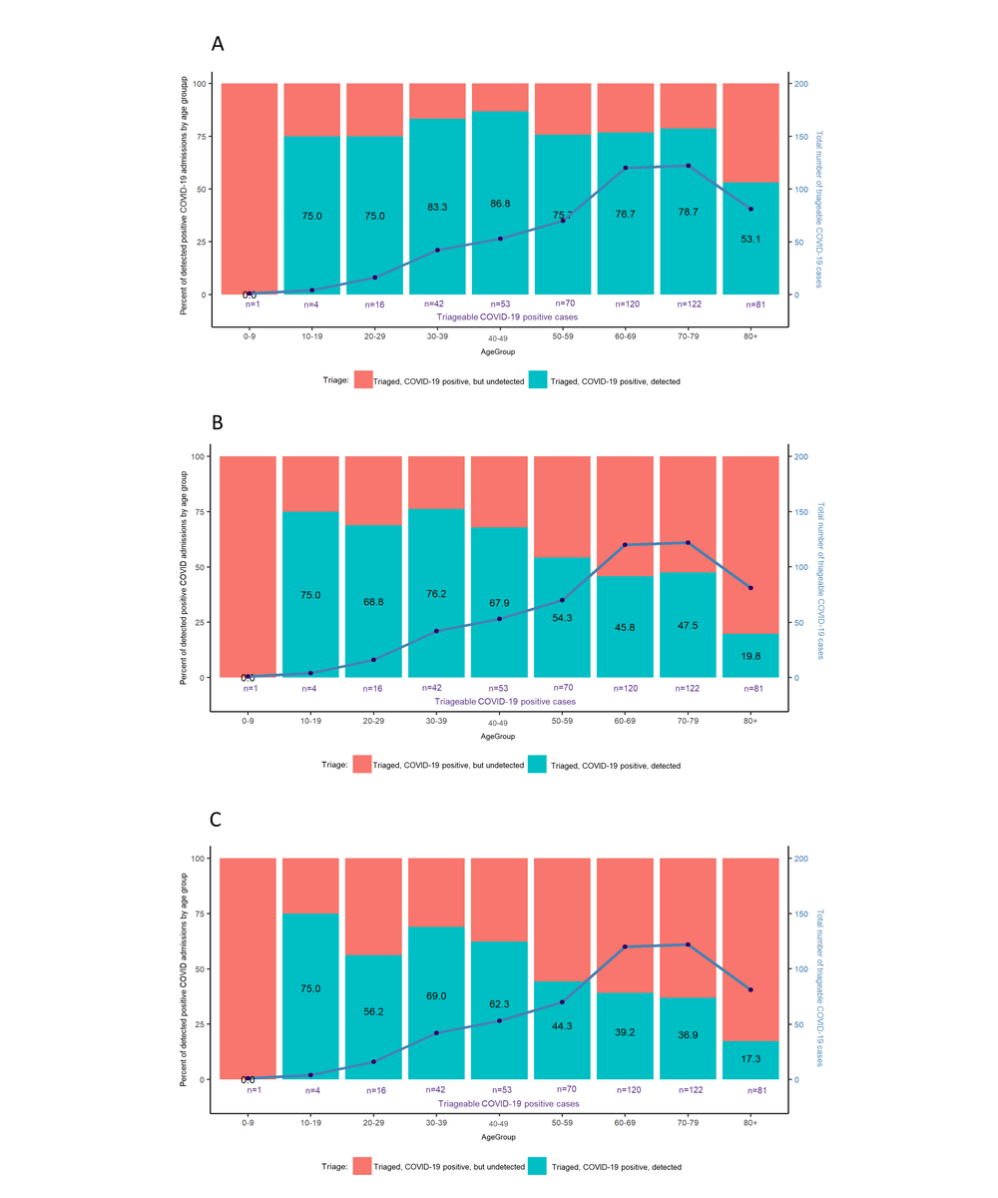
Comparing SARI definitions: patients with COVID-19 detected by age group from February 1 to November 30, 2021. Percent of patients who are COVID-19 positive detected by triage using (A) Maltese SARI "confirmed" and "possible" definitions, (B) Maltese SARI "confirmed" definition, and (C) the World Health Organization's definition. SARI: severe acute respiratory infections.

Figures 4 and 5 compare the current definition we use for SARI, which includes both “confirmed” and “possible” cases adopted by the Maltese team. In contrast, the “confirmed” SARI panel compares “confirmed” SARI cases to the WHO definition by month and age group. Our SARI case definition, including both “confirmed” and “possible” cases, identified substantially more cases, with twice as many patients with COVID-19 detected over the winter months compared to the WHO SARI case definition. However, the “confirmed” cases by themselves, although still an improvement from the WHO SARI case definition, improved at most only 5-15 percentage points (Figure 4). Figure 5 consistently shows a similar pattern for the definition including both “confirmed” and “possible” cases compared to the WHO definition. However, with “confirmed” cases alone, the improvement was only of 5-10 percentage points higher than the WHO definition. (Figure 5).

### Surveillance Outputs

The SARI surveillance team sends case-based and aggregate data locally on a weekly and monthly basis. We also submit accumulated SARI surveillance data to the ECDC. Currently, weekly and monthly data are sent to the ECDC and pooled for specific studies for analysis, including the I-MOVE-COVID-19 study for vaccine effectiveness [11-13].

Data collected through SARI surveillance included extensive COVID-19 data, allowing Malta to conduct its vaccine effectiveness study. Besides the periodic data, our team has also generated:

- 7 monthly SARI reports
- 8 monthly vaccine effectiveness reports
- 10 weekly SARI reports

As a team, we regularly meet stakeholders who we invite to monthly SARI meetings, where ideas for collaboration, improvements to the system, and research outputs are discussed. Additionally, these reports and outputs serve to inform public health management and policy in a dynamic manner [14], informing the government on current respiratory illness outbreaks and local vaccine effectiveness. Currently, vaccine effectiveness against both infection and hospitalization are being analyzed to inform the Ministry of Health on the success of COVID-19 vaccination campaigns. This information will also advise government policy on COVID-19 booster doses in the coming months and years. In addition, E-SARI-Net countries meet monthly to discuss challenges they are facing in the process and adjust the international protocols; these meetings also offer spaces where ideas and experiences are shared for countries to learn from one another’s challenges and adopt a different approach in their work.

## Discussion

### Building Bridges

Setting up a digital, standardized SARI surveillance system was challenging, considering that Malta has gone from paper-based data entry to digitizing its emergency department medical notes within 3 years. However, the biggest challenge to this setup was primarily mapping and identifying key stakeholders and bringing people together amid a busy schedule due to the COVID-19 pandemic under way.

The SARI surveillance team in Malta delivers weekly and monthly reports on SARI to interested stakeholders within Malta’s health system, with monthly meetings to discuss emerging concerns in respiratory illness. These meetings have been key to the program’s success: a space where ideas for data and surveillance processes are discussed and stakeholders are listened to, with the aim of constant improvements to the system. Additionally, reports and meetings also inform decision makers on the policies they can adapt based on the data, which is the key aim of SARI surveillance.

### Principal Findings

#### Demographics

First, there is a gendered difference among patients with SARI, with more male patients (531/903, 58.8%) entering the hospital than female patients (369/903, 40.9%). This difference is more pronounced for patients who are COVID-19 positive, where men (244/380, 64.2%) are hospitalized in larger numbers than women (136/380, 35.8%). Although gender has an association with COVID-19 incidence, the severity of COVID-19 presentation differs according to gender, but the reasons for this difference have yet to be fully explained [15,16]. Second, median age differences for severity categories can be explained by policies at MDH. Those in the older age groups are less likely to be admitted for intensive care, given limited bed space and lower chances of positive outcomes from intensive care. Consequently, the median age of those admitted to intensive care is lower than all hospitalized for SARI.

#### Adoption of the Maltese SARI “Hybrid” Definition

The commencement of SARI surveillance coincided with the worst wave (at the time) of the COVID-19 pandemic in Malta. At first, Malta used the WHO SARI definition in its basis for the inclusion of patients [8]. Following numerous discussions among countries participating in the E-SARI-Net, we expanded its inclusion criteria to include feverishness and shortness of breath. Although an improvement over the WHO criteria, symptoms-based triage inclusion is only marginally better than the WHO SARI definition as compared to the comprehensive system our team has adopted, which heavily relies on clinical diagnosis. This can point to either (1) clinician bias, where COVID-19 cases are more likely to be registered as SARI; (2) poor symptom reporting; or (3) both. Clinician bias is highly likely, especially during the winter months (from February to March and in November 2021) when Malta experienced a COVID-19 wave. This form of bias, called “availability bias,” is one that occurs where a particular diagnosis is given due to the likelihood of it being assigned, given the circumstances or presence in mind [17]. Poor symptom reporting is also increasingly likely during winter months when, under time pressure, clinicians might take a shortened account of symptom history. In the case of older age groups, the observed variability can be attributed to the broad spectrum of COVID-19 symptom presentations or poor patient communication [18]. These findings further motivate the shift to a register-based surveillance system, where symptom reporting will be mandatory, which will likely improve COVID-19 case detection.

#### Comparison With Prior SARI Surveillance

Compared to prior SARI surveillance carried out in Malta, the current system is increasingly comprehensive, including data pertaining to laboratory data, vaccination status, and severity of outcome, among other parameters. This improves the capacity for dynamic decision-making by public health policy makers while allowing Malta to carry out other analysis, such as vaccine effectiveness studies, which were not possible before. Additionally, the current form of surveillance allows Malta to share its data on a European level, for pooled analysis.

Although syndromic parameters are still extracted manually, Malta is aiming to digitize the system in the first half of 2022. The shift of the IT system at MDH to a register-based system is being piloted now and will allow automated extraction of symptom and comorbidity data. This last step will allow us to fully automate our last remaining manual extraction point (symptoms and comorbidities). Through precomposed and easily upgradeable R scripts, this would make SARI surveillance increasingly sustainable [19]. Consequently, we expect the volume of patients with SARI registered in the database to increase substantially as more accurate data are provided, given that currently symptoms are only mentioned at the clinician’s discretion.

The SARI surveillance team has recently incorporated a new stakeholder: the Influenza vaccination team. These data will be included in pooled international influenza vaccine effectiveness studies, such as I-MOVE [20,21]. The collection of treatment data (and resulting outcomes) would also be a positive addition to the surveillance program. This could greatly expand Malta’s research potential, permitting cohort studies or case-control studies to examine the benefit and effectiveness of treatment provided for the patient.

### Strengths and Limitations

Our current system has limitations. First, we only triage patients assigned a provisional diagnosis by clinicians. This will be addressed by the shift to register-based surveillance with mandatory symptomatic entry of SARI-defining symptoms. Second, patients provisionally diagnosed as “SARI” were automatically entered into the database. Among these patients, those who did not meet the triage definition were classified as “possible” SARI cases, whereas all patients meeting SARI definitions were classified as “confirmed.” This was an important factor in recruitment—due to the novelty of the system, urgency of data input on busy days at the emergency department, and the “free-text” nature of entering data, specific symptoms useful for accurate SARI triage might have been missing (eg, fever or cough). These limitations are Malta’s primary motivator to move toward register-based surveillance [22], where the entry of SARI diagnostic criteria would be mandatory at the patient-admission level and extraction is symptoms-based.

However, our system has many strengths. Over the past few months, it has fostered a spirit of cooperation and collaboration among various departments or teams that might have been too busy to collaborate otherwise. This essential outcome is often overlooked but is very much at the core of a surveillance system. Without stakeholders’ trust, neither data provision nor its output would be possible. This is a very positive outcome, and its success might spur further collaboration among different stakeholders that could substantially improve Malta’s public health capacity. We also managed to run a system with few resources, which will ensure long-term sustainability.

### Conclusion

Since February 1, 2021, Malta has successfully established an SARI surveillance system. Although there is much room for improvement, its outcomes substantially improve Malta’s ability to monitor patients with SARI, informing decision-making processes. This is even more important in the context of the COVID-19 pandemic, which was an essential driver in establishing SARI surveillance. Future changes, including the shift to register-based surveillance, should substantially increase both the catchment of SARI as well as our capacity to detect and respond to new disease threats. The establishment of the SARI surveillance system also encourages the development of dynamic policy-making and public health interventions, which can be more effective both on a population level and can make a more efficient use of public health resources. The findings of this study also encourage the examination of the standard WHO definitions of SARI for surveillance teams, who may choose to adapt or expand the definition of SARI based on the context of public health data available to them for better catchment.

## Appendix

Multimedia Appendix 1 List of data collected for Malta’s SARI surveillance system, including possible values and sources.

## Abbreviations

E-SARI-Net: European SARI Network
ECDC: European Centre for Disease Prevention and Control
ICU: intensive care unit
MDH: Mater Dei Hospital
SARI: severe acute respiratory infections
WHO: World Health Organization
