# Supplementary material for: Digitalizing and Upgrading Severe Acute Respiratory Infections Surveillance in Malta: System Development
Source: JMIR Public Health Surveill. 2022 Dec 5;8(12):e37669. doi: 10.2196/37669 (PMC9764153; doi:10.2196/37669)
Supplement: Multimedia Appendix 1 [file publichealth_v8i12e37669_app1.docx]

**Table S1:** List of data collected for Malta’s SARI surveillance system, including possible values and sources

| **Category** | **Variable** | **Values** | **Source** |
| --- | --- | --- | --- |
| Demographics | Age | 0-115 years | Hospital system data |
|  | Gender | Male, female, unknown | Hospital system data |
|  | Care level | Long term care facility, private residence not dependent, private residence dependent, private residence unknown care dependency, unknown care level and residency | Hospital: admission notes; List provided by Ministry for Health |
|  | Health care worker | Yes, no, unknown | Hospital: admission notes; List provided by Ministry for Health |
| Previous hospitalisations | Count of hospital admissions within 365 days | Numeric | Hospital system data: visit history |
|  | Date of most recent admission | Date |  |
| Episode date | Date entered AE | Date |  |
|  | Date of admission to ward/bed assigned | Date |  |
| Outcome | Date of discharge | Date |  |
|  | Date of death | Date |  |
|  | Outcome | Discharge, death, still being treated |  |
| Provisional diagnosis | Provisional diagnosis | S. SARI  B. RESPIRATORY DYSPNOEA  B. RESPIRATORY PNEUMONIA B. RESPIRATORY ASPIRATION PNEUMONIA B. RESPIRATORY SUSPECTED TB B. RESPIRATORY LRTI | Hospital system data |
| Treatment ICU | Date entered ICU | Date | Hospital system data: visit history |
|  | Date left ICU | Date | Hospital system data: visit history |
| Treatment | Respiratory oxygen, ventilator, antiviral prophylaxis, antiviral medication, antiviral treatment | Yes, no, unknown | Hospital: Admission notes and discharge letters |
|  | Antiviral prophylaxis date, antiviral medication date | Date | Hospital: discharge letters |
| Symptoms | Symptom onset date | Date | Hospital: admission notes |
|  | Fever (38.0C° =<), feverishness (37.0C° – 37.9C°), cough, shortness of breath, diarrhoea, vomiting, nausea, chest pain, abdominal pain, coryza, sore throat, anosmia, loss of taste, altered taste, malaise, headache, myalgia, deterioration of general condition | Yes, no, unknown | Hospital: admission notes |
|  | Other symptoms | List of all other reported symptoms (string) | Hospital: admission notes |
| Underlying conditions | Asthma, diabetes, HIV, obesity, dementia, hypertension, cancer, CHD, immunodeficient, kidney disease, liver disease, chronic lung disease, neurocognitive, neuromuscular | Yes, no, unknown | Hospital: admission notes |
|  | Smoking history | Never smoked, former smoker (stopped smoking at least 1 year before inclusion in the study), current smoker, unknown | Hospital: admission notes |
|  | Pregnancy status | Unknown, not pregnant, pregnancy trimester, not applicable | Hospital: admission notes |
|  | Other underlying conditions | List of all other underlying medical conditions (string) | Hospital: admission notes |
| Clinical diagnosis | Pneumonia, ARDS, bronchiolitis, encephalitis, myocarditis, sepsis | Yes, no, unknown | Hospital: admission notes and discharge letters |
|  | Other clinic | List of all other clinical diagnosis (string) | Hospital: admission notes and discharge letters |
| Testing results for respiratory pathogens | Testing results for Covid-19 | Date of swab and result (Detected/Not detected) | Hospital laboratory data; Private PCR, rapid antigen testing database |
|  | Testing results for other respiratory pathogens | Date of swab, test type and result (Detected/Not detected) | Hospital laboratory data |
|  | Covid-19 Sequencing results | Clade | Hospital laboratory data |
| Vaccination | Dates of vaccination against influenza | Date | Public Health of Malta vaccination data |
|  | Dates of vaccination against Covid-19 | Date | Hospital: Covid-19 vaccination database |
|  | Vaccine brand | Pfizer/BioNTech (Comirnaty),  AstraZeneca (Vaxzevria),  Moderna (Spikevax),  Johnson & Johnson (Janssen) | Hospital: Covid-19 vaccination database |
|  | Vaccine batch | Various (string) | Hospital: Covid-19 vaccination database |
|  | Number of vaccine doses | Numeric | Hospital: Covid-19 vaccination database |
| All-cause admissions | All-cause weekly admissions by age groups | Numeric | Hospital census |
| Population data | Malta’s Population data by age groups | Numeric | Ministry for Health |
